# The impact of microbiome diversity and composition on host health and susceptibility to disease across amphibian life stages

**DOI:** 10.3389/famrs.2025.1666714

**Published:** 2026-01-28

**Authors:** Abigail J. Miller, Jamie Voyles

**Affiliations:** Department of Biology, University of Nevada, Reno, Reno, NV, United States

**Keywords:** amphibian, health, disease susceptibility, Batrachochytrium dendrobatidis, microbiome, gnotobiotic

## Abstract

Germ-free (i.e., absence of all microbes) and gnotobiotic (i.e., specific known microbial communities) study systems have classically been used to investigate the critical role the microbiome plays in the development of the host immune system. To date, most systems that have been used to experimentally manipulate the microbiome have been developed in model organisms, such as mice and pigs. However, amphibians are rapidly emerging as a valuable model for studying host–microbiome interactions and their effects on health and immunity, especially in the context of infectious disease. Amphibians are a particularly compelling system because, unlike many current systems, they include many species that do not need direct parental care during development, and they undergo a complete reorganization of their immune system during metamorphosis. Amphibians are a group of particular conservation importance as they are currently affected by the infectious disease chytridiomycosis, caused by the fungal pathogen *Batrachochytrium dendrobatidis*. Here, we review current research aimed at manipulating the amphibian microbiome through the use of antimicrobial treatments, with a focus on how a depletion of the microbiome diversity influences host development, immunity, and susceptibility to infectious disease. We structure our review in three parts: (1) how microbiome depletion affects chytridiomycosis disease dynamics, (2) how microbiome bioaugmentation through the use of probiotics influences susceptibility to chytridiomycosis, and (3) how microbiome depletion affects amphibian health across different life stages. Overall, research on this topic is important for the conservation of wild amphibian populations because it adds to understanding of the amphibian immune system and susceptibility to disease, both of which are important to inform management strategies and optimize potential therapeutics to aid susceptible populations.

## Introduction

The microbiome plays an essential role in the development and maintenance of the host immune system over the course of life (Thomas et al., 2017; Dominguez-Bello et al., 2019). During development, bacteria-associated molecules can aid in the development of immunity through interactions with Toll-like receptors, thereby priming immune responses for later life (Dominguez-Bello et al., 2019). Then, in later life stages, the microbiome is known to be involved in many immune functions, such as stimulating the production of immune cells and cytokines (Lambring et al., 2019). Disruptions to the microbiome that occur both during and after development have been connected to a multitude of inflammatory disorders, such as inflammatory bowel disease (Lambring et al., 2019).

Germ-free (i.e., absence of all microbes) and gnotobiotic (i.e., specific known microbial communities) systems have been crucial for understanding how the microbiome affects host immunity (Gensollen et al., 2016; Lambring et al., 2019). For example, mice that develop without a microbiome are known to have underdeveloped lymphoid tissues (i.e., smaller Peyer’s patches) and less antimicrobial peptides in their intestine (Gensollen et al., 2016). Additionally, most research on the importance of the microbiome to date has been accomplished using animal model germ-free systems (e.g., mice, rats, pigs; Konig and Markl, 1987; Andersen et al., 2011). However, the microbiome is also known to be important in non-model systems, such as in amphibians (Rebollar et al., 2020). Amphibians have the potential to be a valuable model for work on the microbiome, especially during development, because, unlike many current systems, they lack a need for direct parental care during development and they undergo a complete reorganization of their immune system during metamorphosis (Konig and Markl, 1987; Tobias et al., 1998; Andersen et al., 2011; Kohl et al., 2013). Amphibians are also a relevant system in a conservation context because they are currently threatened by the infectious disease chytridiomycosis, which is caused by the fungal pathogen *Batrachochytrium dendrobatidis (Bd*; Berger et al., 1998; Longcore et al., 1999; Scheele et al., 2019).

Amphibian species vary in their responses to chytridiomycosis, and the microbiome has been implicated in differential susceptibility (Rebollar et al., 2016, Rebollar et al., 2020). Previous studies on chytridiomycosis in wild populations have shown an inverse correlation between the abundance of bacteria in the cutaneous microbiome capable of inhibiting the growth of *Bd* and population declines due to *Bd* (i.e., populations that have a higher presence of *Bd*-inhibitory bacteria in their skin microbiomes tended to have less severe declines due to *Bd*; Grogan et al., 2018; Rebollar et al., 2020). Further, there are specific candidate probiotic microbes that have protective effects against chytridiomycosis (Harris et al., 2009). The most widely used probiotic bacteria in studies centered on decreasing susceptibility to *Bd* is *Janthinobacterium lividum* (hereafter, *J. lividum*), which is a member of the phylum Pseudomonadota (previously Proteobacteria; Kueneman et al., 2016). Along with *in vitro* protective effects, it has also been shown to offer protection to some species of amphibians *in vivo* through the creation of secondary metabolites (i.e., violacein; Rebollar et al., 2020). Together, this body of research positions amphibians as a strong emerging model system to study the impacts of the microbiome on health, development, and disease in the broader context of conservation, infectious disease, and wildlife management (Rebollar et al., 2020).

Although the microbiome is undoubtedly important, we are still investigating its role in amphibian health and disease (Becker et al., 2011; Kearns et al., 2017; Rebollar et al., 2020). Previous work on microbes and amphibians has focused on identifying *Bd*-inhibitory bacteria to understand how they may be associated with disease susceptibility in different species (Woodhams et al., 2015; Grogan et al., 2018; Rebollar et al., 2020). More recently, to better understand the microbiome’s full influence on amphibian health and disease, researchers are beginning to develop germ-free and gnotobiotic systems in amphibians (Harris et al., 2009; Becker and Harris, 2010; Knutie et al., 2017; Warne et al., 2019; Miller et al., 2023). These studies are aimed at manipulating the diversity and composition of the microbiome at different life stages and in the context of different infectious diseases (Harris et al., 2009; Becker and Harris, 2010; Knutie et al., 2017; Warne et al., 2019; Miller et al., 2023). This work is important because germ-free systems are a historic innovation that has allowed us to see a more complete picture of the role the microbiome plays in host development and overall function (Yi and Li, 2012). Germ-free and gnotobiotic systems have also allowed us to understand how specific, individual microbes alter host health in ways that had not been possible before their creation (Yi and Li, 2012).

The development of a germ-free amphibian system could answer multiple questions surrounding amphibian development and disease. First, how does development in the absence of microbes affect both amphibian innate and adaptive immunity? This question is especially applicable as we know certain aspects of amphibian immunity are highly influenced by interactions with commensal microbes (i.e., amphibian skin secretions; Rollins-Smith, 2005, Rollins-Smith, 2023; Flechas et al., 2019; Woodhams et al., 2020; Brunetti et al., 2022). Additionally, this system could answer questions relating to how the microbiome shapes metamorphosis, relevant because metamorphosis is an important influence on microbiome assembly (Kohl et al., 2013; Longo et al., 2015; Martínez-Ugalde et al., 2022; Hartmann et al., 2023). Germ-free amphibian systems would therefore allow us to completely decouple immunity and host development from the influence of the microbiome, enabling understanding of its full impact in a standardized and context-independent way that is not possible without gnotobiotics. Finally, the generation of a germ-free amphibian model would allow for comparisons across model and non-model organisms that would help elucidate trends in the microbiome’s influence on a broad scale (Yi and Li, 2012).

Previous reviews have focused on the microbiome’s overall involvement in amphibian disease dynamics (Rebollar et al., 2020). Here, we build off this background, providing a narrower focus on the current research on (1) how microbiome depletion affects chytridiomycosis disease dynamics, (2) how microbiome bioaugmentation through probiotics influences susceptibility to chytridiomycosis, and (3) how microbiome depletion affects amphibian health across different life stages. To identify the existing literature on these narrower topics, we refined definitions to understand the available data. For example, we defined “microbial depletion” as a disruption to the microbiome that decreases microbial richness and abundances (e.g., administration of antimicrobials) and a “disruption to the microbiome” as an event that alters microbiome composition (but one that does not have to reduce the alpha diversity). We review studies involving microbiome depletion, bioaugmentation, and impacts across life stages because they are three related topics that are significant to the generation of germ-free and gnotobiotic amphibians, a relatively new focal study area, and a topic of utmost conservation importance.

## Chytridiomycosis and microbial depletion

The microbiome is a known factor influencing susceptibility to chytridiomycosis, and recent studies have contributed to our knowledge of its influence on amphibian disease dynamics (Rebollar et al., 2020; Ruthsatz et al., 2020; Nava-González et al., 2021; Jiménez et al., 2022; Buttimer et al., 2024). Studies involving salamander (*Ambystoma rivulare, Plethodon cinereus, Eurcyea bislineata, Desmognathus monticola*, and *Notophthalmus viridescens*) and frog (*Rana [Lithobates] spectabilis*) species have further emphasized that overall bacterial richness, as well as specifically *Bd*-inhibitory richness of the cutaneous microbiome, is often associated with lower pathogen loads and infection prevalence in this system (Nava-González et al., 2021; Jiménez et al., 2022). The environment can also alter the microbiome in ways that affect *Bd* susceptibility, as studies have found drought conditions can lead to less *Bd*-inhibitory bacteria in the skin microbiome, potentially increasing susceptibility to *Bd* in some species (*Brachycephalus rotenbergae*;Buttimer et al., 2024). In contrast to this, Ruthsatz et al. (2020) found a weak positive correlation between infection intensity and bacterial richness in infected treefrogs (*Hylidae and Phyllomedusidae*) and posit that this pattern is related to the low pathogen load observed in species in this study (Ruthsatz et al., 2020). Overall, these studies suggest that the microbiome influences chytridiomycosis susceptibility, but the specific mechanisms of how the microbiome alters susceptibility remain unresolved and warrant further investigation.

The microbiome can affect chytridiomycosis disease dynamics, and, conversely, *Bd* infection can affect the microbiome (Jani and Briggs, 2014; Jani et al., 2021). Both laboratory and field studies have shown *Bd* can alter bacterial community composition (Jani and Briggs, 2014). Jani et al. (2021) found infection changed the composition of the mountain yellow-legged frog (*Rana [Lithobates] muscosa*) skin microbiome, and subsequent clearing of infection with an antifungal treatment (i.e., itraconazole) was not sufficient to return skin microbiome composition or abundances to pre-infection structure, indicating a low resilience following a disease disturbance (Jani et al., 2021). In contrast, southern leopard frogs (*Rana [Lithobates] sphenocephalus*) first exposed to a biopesticidal bacteria (i.e., *Bacillus thuringiensis*) and then exposed to *Bd* had a skin microbiome able to recover able to recover after both disturbances, which suggests the microbiome in this context is resilient and might contribute to the lack of population declines in this species (Weeks et al., 2020). This recent body of research has highlighted the complicated and often both species-specific and context-dependent relationship between microbes and *Bd*, and the need for amphibian germ-free systems to be able to isolate and understand microbial effects (Ruthsatz et al., 2020; Weeks et al., 2020; Jani et al., 2021; Nava-González et al., 2021; Jiménez et al., 2022).

In the context of generating germ-reduced amphibians, studies have shown that depleting the diversity of the microbiome can increase chytridiomycosis susceptibility (Becker and Harris, 2010; Woodhams et al., 2012; Holden et al., 2015). Depleting the microbiome through the use of antimicrobials before exposing adult amphibians to *Bd* may increase pathogen load (Holden et al., 2015). For example, Holden et al. (2015) depleted the microbiome of post-metamorphic southern leopard froglets (*Rana [Lithobates] sphenocephala*) with an antibiotic cocktail, challenged them with *Bd*, and subsequently found that the antibiotic-treated froglets had an increased *Bd* load compared to control froglets early in infection (Holden et al., 2015). They also saw *Bd* load on the day of death was higher in antibiotic-treated froglets that succumbed to infection, although they found no significant differences in survival among groups (Holden et al., 2015).

Microbial depletion can also increase clinical signs of chytridiomycosis in infected amphibians (Becker and Harris, 2010; Woodhams et al., 2012). Redback salamanders (*Plethodon cinereus*) treated with antibiotics and then exposed to *Bd* displayed increased limb-lifting behavior, a clinical sign of *Bd* infection, as well as had a greater loss of body mass compared to salamanders with intact microbiomes (Becker and Harris, 2010). Additionally, the microbiome seems to work synergistically with host skin immune defenses during infection, as there are examples of host defenses increasing during infection to compensate for the loss of inhibitory potential of the microbiome caused by a depletion (Woodhams et al., 2012). For example, previous studies have shown that European water frogs (*Pelophylax esculentus* and *P. lessonae*) with antibiotic-depleted microbiomes had increased production of antimicrobial peptides when exposed to *Bd*, and antibiotic-treated frogs had different peptide profiles compared to the frogs with intact microbiomes (Woodhams et al., 2012). Together, these results suggest microbiome depletion may generally increase susceptibility to chytridiomycosis, and/or mediate immune responses to *Bd*, offering further evidence to suggest the diversity of the microbiome is a key driver of chytridiomycosis disease dynamics (Becker and Harris, 2010; Woodhams et al., 2012).

## Bioaugmentation in amphibians

A depletion of the amphibian microbiome can alter host immune responses and increase susceptibility to disease (Becker and Harris, 2010; Woodhams et al., 2012; Holden et al., 2015). Conversely, bioaugmentation (i.e., the addition of microbes to a system) of the amphibian microbiome with probiotic bacteria may bolster the microbiome’s protection against disease, especially within the *Bd* disease system (Harris et al., 2009; Kueneman et al., 2016). The probiotic bacteria *J. lividum* has primarily been used to decrease susceptibility to chytridiomycosis, with administration of *J. lividum* prior to exposure to *Bd* increasing survival, preventing *Bd*-induced weight loss, and decreasing pathogen loads in infected mountain yellow-legged frogs (*Rana [Lithobates] muscosa*; Harris et al., 2009). However, administration of *J. lividum* does not always decrease susceptibility to *Bd* in susceptible amphibian species (Becker et al., 2011). In Panamanian golden frogs (*Atelopus zeteki*), for example, *J. lividum* exposure did not lead to any differences in survival or in *Bd* loads among treatment groups that were either exposed to the probiotic or not throughout the infection (Becker et al., 2011). Researchers also saw initial success in establishment of *J. lividum* on the frog skin, but over the course of the experiment the amount of *J. lividum* steadily decreased, potentially contributing to the lack of protective effects (Becker et al., 2011). Similarly, treatment of sub-adult Sierra Nevada yellow-legged frogs (*Rana [Lithobates] sierrae*) with *J. lividum* did not offer protective effects against *Bd*, and the probiotic similarly did not successfully establish on frog skin (Knapp et al., 2022). These studies highlight the importance of research on bioaugmentation in the *Bd* system. Due to the complex nature of the effect of probiotics on disease dynamics, these investigations indicate that more research is needed to optimize *J. lividum* as a probiotic *in vivo* (Harris et al., 2009; Becker et al., 2011; Knapp et al., 2022).

Previous work has also assessed whether adding a combination of different probiotic (i.e., *Bd-inhibitory*) bacteria amplifies potential protective effects (Woodhams et al., 2020; Becker et al., 2021). Becker et al. (2021) tested two probiotic treatments: a single bacterium (*Diaphorobacter* sp.) genetically modified to produce violacein and a consortium of nine different microbial isolates capable of inhibiting *Bd* growth in culture (Becker et al., 2021). However, neither of these treatments increased survival in Panamanian golden frogs (*Atelopus zeteki*) that were exposed to *Bd*, and the genetically modified bacterial treatment was undetectable on frogs four weeks after initial exposure (Becker et al., 2021). Woodhams et al. (2020) also applied a consortium of four different *Bd*-inhibitory bacteria (*Janthinobacterium lividum*, *Chryseobacterium ureilyticum*, *Serratia grimesii*, and *Pseudomonas* sp.) to Sierra Nevada yellow-legged frogs (*Rana [Lithobates] sierrae*) and similarly did not find persistence of the probiotic mixture on frog skin or any survival effects when exposed to *Bd* (Woodhams et al., 2020). These studies, paired with the results from Becker et al. (2011), highlight an important caveat of probiotics as a viable treatment for wildlife disease (Becker et al., 2011, 2021; Woodhams et al., 2020; Knapp et al., 2022). Namely, probiotics are a compelling treatment because of their potential to offer longterm effects on susceptibility to disease (Harris et al., 2009), but there are challenges in establishing probiotic persistence in multiple amphibian species (Becker et al., 2011, Becker et al., 2021; Woodhams et al., 2020; Knapp et al., 2022). These contrasting results, and the potential of probiotics as a treatment, suggest a need for more work on ways to increase successful establishment and persistence (Becker and Harris, 2010; Becker et al., 2011, Becker et al., 2021; Holden et al., 2015; Woodhams et al., 2020; Knapp et al., 2022). For example, future research on probiotics and *Bd* could focus on increasing persistence through the use of prebiotics or selective antibiotic treatments before probiotic exposure (McKenzie et al., 2018).

Studies have also combined both the concepts of bioaugmentation and microbiome depletion (Kueneman et al., 2016). Kueneman et al. (2016) designed an experiment to address how the diversity of the amphibian microbiome is affected by captivity and how augmentation with probiotics can potentially rescue the detrimental effects of captivity on the microbiome (Kueneman et al., 2016). They first measured the composition and diversity of the skin microbiome over time in captive boreal toads (*Anaxyrus boreas*), and compared this to the microbiomes of wild toads (Kueneman et al., 2016). Toads in captivity had a less diverse skin microbiome and, more importantly, the *Bd*-inhibitory bacteria present in the toad microbiome decreased over time, which was correlated with an increase in susceptibility to *Bd* (Kueneman et al., 2016). In addition, a subset of the toads were exposed to the *Bd*-inhibitory bacterium *J. lividum* and then re-exposed to *Bd*, resulting in an increase in survival with the addition of *J. lividum* (Kueneman et al., 2016). Complementary to this study, Estrada et al. (2022) found that limosa harlequin frogs (*Atelopus limosus*) bred in captivity and then released into the wild showed an increase in skin microbiome diversity, with microbiome diversity increasingly resembling the microbiomes of wild *A. limosus* after only 27 days (Estrada et al., 2022). Other studies have observed similar results, namely that there are differences in captive amphibian microbiomes (i.e., skin, gut, and mouth microbiomes) and that, upon introduction to wild conditions, the captive microbiome mirrors that, of wild amphibians (i.e., microbiome rewilding; Kueneman et al., 2022; Korpita et al., 2023). Most relevant, the antifungal function of captive-bred frog (*Atelopus varius*) skin microbiomes has been shown to increase in wild conditions (Kueneman et al., 2022). Together, these studies emphasize the important effects of captivity on the diversity of the microbiome in amphibians, which can contribute to susceptibility to *Bd* (Kueneman et al., 2016, Kueneman et al., 2022; Estrada et al., 2022; Korpita et al., 2023). Overall, probiotics and bioaugmentation stand as compelling potential therapeutic tools to aid in the recovery and protection of wild populations against chytridiomycosis. They may also increase the success of reintroduction of captive-bred amphibians into wild conditions (Harris et al., 2009; Kueneman et al., 2016, Kueneman et al., 2022).

## Disruptions to the amphibian microbiome across life stages

Amphibians are a valuable emerging model system to study the microbiome in part because many species have both aquatic and terrestrial life stages, and metamorphosis is an important influence on microbiome structure (Kohl et al., 2013; Longo et al., 2015; Martínez-Ugalde et al., 2022; Hartmann et al., 2023). In indirect developing amphibians (e.g., *Rana [Lithobates] pipiens*), the larval gut microbiome composition has been noted as being more similar to aquatic species such as fish, where the adult gut microbiome is more similar to amniotic species (Kohl et al., 2013). Restructuring of the gut microbiome during metamorphosis has been linked to both differences in diet and in gut morphologies between larval and adult amphibians (Chai et al., 2018). In addition to indirect development with discrete larval and adult stages, amphibians can also have a number of unique developmental modes (Longo et al., 2015; Martínez-Ugalde et al., 2022; Hartmann et al., 2023). However, comparable to indirect developing amphibians, early life stages of both direct developing species and paedomorphic (i.e., species that retain larval features) species have been shown to have cutaneous microbiomes that differ from adults in terms of diversity and composition (Longo et al., 2015; Martínez-Ugalde et al., 2022; Hartmann et al., 2023). Differences in microbiome composition and diversity between early and late life stages may also be related to immune development and function (Kohl et al., 2013; Longo et al., 2015; Martínez-Ugalde et al., 2022). For example, previous studies have posited that adult amphibians have more developed immune systems, which may be a stronger selective pressure on the composition of the microbiome, resulting in the differences we see between larval and adult life stages (Kohl et al., 2013; Longo et al., 2015).

The larval microbiome often differs from the microbiome of adult amphibians, however, it can affect disease susceptibility in similar ways (Siomko et al., 2023; Loyau et al., 2024). Loyau et al. (2024) compared the tadpole skin microbiome of three cohabitating species (*Alytes obstetricans, Rana [Lithobates] temporaria*, and *Bufo spinosus*) differentially susceptible to *Bd* and found the lowest relative abundance of *Bd*-inhibitory bacteria in the most susceptible species (i.e., *Alytes obstetricans*; Loyau et al., 2024). Researchers have also found prophylactic exposure of *Bd* metabolites to tadpoles (*Pseudacris regilla*) can increase the abundance of *Bd*-inhibitory bacteria in the tadpole skin microbiome (Siomko et al., 2023). Collectively, these studies show that although the microbiome differs in composition and diversity between larval and adult amphibians, the relationship between the microbiome and *Bd*, especially regarding *Bd*-inhibitory bacteria, seems to transcend life stage (Kohl et al., 2013; Siomko et al., 2023; Loyau et al., 2024).

Because development and metamorphosis are key stages for microbiome assembly in amphibians, disruptions to the microbiome during development can have especially large and lasting impacts on amphibian health (Kohl et al., 2013; Longo et al., 2015; Knutie et al., 2017). Multiple studies suggest that a depletion of the larval microbiome can affect growth, time to metamorphosis, and survival during development (Knutie et al., 2017; Warne et al., 2019; Emerson and Woodley, 2024). Northern leopard frog (*Rana [Lithobates] pipiens*) tadpoles reared in sterile water to decrease the diversity of their microbiome were 20-25% larger than tadpoles with intact microbiomes (Emerson and Woodley, 2024). Tadpoles reared in sterile water also had different brain morphologies, with a larger overall brain mass, but a smaller width of the medulla, suggesting the diversity of the microbiome is potentially correlated with healthy brain development in amphibians (Emerson and Woodley, 2024). Tadpoles of various species (i.e., *Rana [Lithobates] sylvaticus* and *Osteopilus septentrionalis*) with depleted microbiomes have also been shown to have a decreased metabolic rate over developmental time and a longer time to metamorphosis (Knutie et al., 2017; Warne et al., 2019). Tadpoles with depleted microbiomes can also have both a lower survival and a higher prevalence of tail malformations during development (Knutie et al., 2017; Warne et al., 2019). The microbiome can therefore influence amphibian health during development, with a depletion of the microbiome having the potential to affect tadpole morphology, metabolic rate, and survival to adulthood (Knutie et al., 2017; Warne et al., 2019; Emerson and Woodley, 2024).

A depletion of the amphibian microbiome during larval life stages can also influence susceptibility to disease both during development and into adulthood (Knutie et al., 2017; Warne et al., 2019). Sterilized wood frog tadpoles (*Rana [Lithobates] sylvaticus*) were more likely to die of ranavirus than tadpoles with intact microbiomes (Warne et al., 2019). There is also evidence of increased disease susceptibility persisting through metamorphosis (Knutie et al., 2017). Cuban tree frogs (*Osteopilus septentrionalis*) that experienced a depletion of their microbiome with antibiotics as tadpoles had a higher percentage of parasitic worms established in the gut after exposure (Knutie et al., 2017). Researchers also found there was no difference in percentage of parasitic worms that initially penetrated the skin of the frogs after exposure, only those that established in the gut (Knutie et al., 2017). These results show a difference in adult frog resistance to infection due to a disruption of the microbiome during the larval stage, suggesting the larval microbiome can influence adult amphibian health (Knutie et al., 2017). However, the larval microbiome does not always affect adult susceptibility to disease (Miller et al., 2023). Miller et al. (2023) depleted the microbiome of African clawed frog (*Xenopus laevis*) tadpoles and reared the tadpoles in sterile conditions until metamorphosis (Miller et al., 2023). After metamorphosis, they exposed the froglets to *Bd* and found no differences in infection patterns (i.e., pathogen load or clinical signs of infection) among the treatment groups (Miller et al., 2023). Comparable to other findings in amphibian microbiome research, this suggests that the effects of a disruption to the larval microbiome that carry through metamorphosis may be both species and disease specific (Miller et al., 2023).

## Conclusion and future directions

Thus far, important work has been done to optimize systems that allow for more precise manipulation of the amphibian microbiome (Becker and Harris, 2010; Woodhams et al., 2012; Holden et al., 2015; Knutie et al., 2017; Warne et al., 2019; Miller et al., 2023). This work has shown both microbial depletion and bioaugmentation with probiotics can play an important role in health and disease in amphibians across life stages (Becker and Harris, 2010; Becker et al., 2011; Holden et al., 2015; Knutie et al., 2017; Warne et al., 2019). Unsurprisingly, the effect of the microbiome on amphibian health is complex, often species and life stage specific, and likely mediated by environmental factors (Jimeénez and Sommer, 2017; Buttimer et al., 2024). Because of the complex and often context-dependent effects of the microbiome on the host, more work towards the generation of germ-free systems is needed to fully isolate and understand the effects of the microbiome on amphibian health and disease susceptibility (Harris et al., 2009; Becker et al., 2011; Knutie et al., 2017; Miller et al., 2023).

Much of the research on the amphibian microbiome has focused on the bacterial microbiome, with a lack of understanding of the collective role other types of microbes play in this system (e.g., viruses and fungi that respectively comprise the virome and mycobiome; Kearns et al., 2017). Because the causative agent of chytridiomycosis is a fungus, understanding the mycobiome may yield important insights for *Bd* research (Kearns et al., 2017; Rebollar et al., 2020). In fact, Kearns et al. (2017) showed that there are members of the amphibian mycobiome that can inhibit the growth of *Bd* and also identified possible candidate probiotic fungi (Kearns et al., 2017). They suggest these potential probiotic fungi may be an even more successful alternative to probiotic bacteria for use in chytridiomycosis research, as they elicit less of a stress response during exposures with frogs (i.e., frogs release less stress hormones during exposure; Kearns et al., 2017). Building off of these findings, McKnight et al. (2022) compared the cutaneous bacterial and fungal microbiome of four different Australian frog species (*Litoria dayi, Litoria nannotis, Litoria serrata*, and *Litoria wilcoxii*) and found the bacterial and fungal microbiomes were correlated in terms of richness and beta diversity (McKnight et al., 2022). They also found both bacterial and fungal richness were correlated with historic *Bd* declines and recoveries (i.e., a high microbial richness was found in species that have historically recovered from *Bd*; McKnight et al., 2022). Given this information, further research addressing how a depletion of the mycobiome as well as bioaugmentation with fungal probiotics affects disease dynamics in the chytridiomycosis system could be compelling (Kearns et al., 2017). It will also be interesting to explore how exposure to different probiotic fungi prior to exposure to a pathogen may prime the immune system and subsequently alter host immune responses during infection (Kearns et al., 2017; Rebollar et al., 2020). Probiotics are a promising emerging therapeutic to aid susceptible amphibian populations not only fighting chytridiomycosis but also in other diseases, such as those caused by ranaviruses. More research here is needed to optimize them as a viable treatment for wild amphibians (Harris et al., 2009; Becker et al., 2011; Kueneman et al., 2016; Kearns et al., 2017).

Another avenue for additional microbiome research in amphibians would be addressing how a disruption to the larval microbiome may have impacts that carry through metamorphosis (Knutie et al., 2017; Miller et al., 2023). It has been suggested that larval microbiome disruptions likely affect the adult microbiome, which may also affect adult host health (Knutie et al., 2017). Further, we are beginning to understand how disruptions to the microbiome may affect disease susceptibility and development in amphibians (i.e., growth, time to metamorphosis, survival; Knutie et al., 2017; Warne et al., 2019; Emerson and Woodley, 2024), but little has been done to understand how the developing immune system is impacted by the microbiome and how that in turn affects adult immunity after metamorphosis (Knutie et al., 2017; Miller et al., 2023). Amphibians restructure their microbiome during metamorphosis, and therefore understanding how a disruption before this restructuring might affect the microbiome after restructuring could yield interesting insights (Kohl et al., 2013). Knutie et al. (2017) began to explore this idea with disease susceptibility in their paper, and further research building off the connection between the larval and adult microbiome in the context of amphibian disease and immune development is important (Knutie et al., 2017).

Additionally, in terms of immune system development, it will be important to know if the amphibian microbiome alters the host immune system in ways that complement or contrast with current germ-free model systems. For example, we know lymphoid tissue development is altered in germ-free mice (Gensollen et al., 2016). Yet little is known about how the development of lymphoid organs might be altered in germ-free amphibians, and if those changes may last through adulthood (Miller et al., 2023). Through studies using African clawed frogs (*Xenopus laevis*), we know larval amphibians develop the thymus and spleen in early life, and these lymphoid organs generate lymphocytes that increase in number until metamorphosis (Rollins-Smith, 1998). At metamorphosis, lymphocyte numbers decline in tadpole lymphoid organs (Rollins-Smith, 1998). The microbiome, or a lack of a microbiome, may alter the lymphocyte developmental process both before and after metamorphosis in amphibians (Miller et al., 2023). Overall, amphibians provide a unique system to study the microbiome and immune development, especially because of the process of metamorphosis. Further research on immune development in germ-free amphibians could open new avenues for immunological research by highlighting possible similarities and differences in the microbiome’s influence on immunity in different model organisms, while also creating important advances for conservation biology.

Another important aspect of amphibian immunity are skin secretions, an innate immune defense that plays a key role against multiple infectious diseases, including chytridiomycosis (Woodhams et al., 2012). Currently, there are papers suggesting the microbiome can work synergistically with the host innate immune system to increase the antimicrobial properties of skin secretions, but little is known about how the secretions might be altered when they develop in a germ-free system (Rollins-Smith, 2005, Rollins-Smith, 2023; Woodhams et al., 2012). For example, the minimum concentration needed to inhibit the growth of *Bd* was lowest when *Bd* was exposed to both metabolites created by the symbiotic bacteria *Pseudomonas fluorescens* and amphibian antimicrobial peptides, as opposed to when *Bd* was exposed to either metabolites or antimicrobial peptides in isolation (Myers et al., 2012). Further, Woodhams et al. (2020) identified a novel antimicrobial peptide (i.e., Brevinin-1Ma) after applying a consortium of *Bd*-inhibitory bacteria to Sierra Nevada yellow-legged frogs (*Rana [Lithobates] sierrae*), which was found to both inhibit the growth of *Bd* and facilitate the growth of multiple *Bd-inhibitory bacteria* (*Janthinobacterium lividum*, *Chryseobacterium ureilyticum*, *Serratia grimesii*, and *Pseudomonas* sp.; Woodhams et al., 2020). Indeed, there are multiple examples of amphibian antimicrobial peptides increasing the growth of bacteria isolated from the skin microbiome, and studies have also shown that certain symbiotic microbes (*Pseudomonas* sp.) have mechanisms to resist inhibition caused by amphibian secretions (Flechas et al., 2019; Woodhams et al., 2020; Brunetti et al., 2022; Rollins-Smith, 2023). Because of this strong relationship between the microbiome and host skin immune defenses, understanding how skin secretions develop, and their subsequent antimicrobial potential without a microbiome, could teach us important lessons about how the microbiome is contributing to interspecific susceptibility (Woodhams et al., 2012, Woodhams et al., 2020; Flechas et al., 2019).

In this review, we have synthesized emerging work towards the generation of germ-free or gnotobiotic amphibian systems that contribute to understanding of the microbiome’s influence on amphibian development, health, and disease dynamics, especially in the *Bd* system. Overall, the current body of research suggests that disruptions to the amphibian microbiome early in life can lead to differences in development, especially in regard to growth and time to metamorphosis (Knutie et al., 2017; Warne et al., 2019; Emerson and Woodley, 2024). Some of these changes can persist after metamorphosis, such as susceptibility to disease in some cases (Knutie et al., 2017). Depleting the amphibian microbiome in adulthood can also affect host health, also leading to changes in disease susceptibility in many cases (Becker and Harris, 2010; Holden et al., 2015). Finally, bioaugmentation is a compelling potential therapeutic to bolster the microbiome and offer protection against disease, and this may be especially relevant in captive breeding contexts (Kueneman et al., 2016, Kueneman et al., 2022; Estrada et al., 2022; Korpita et al., 2023). However, the results of bioaugmentation can vary from one species to the next (Harris et al., 2009; Becker et al., 2011; Kueneman et al., 2016). More research is needed to understand how both depleting the microbiome as well as bioaugmenting may alter the development of the immune system in amphibians and host immune responses during infection. Amphibians differ from current model systems because of developmental mode, development decoupled from parental care, and the restructuring of both the microbiome and the immune system during metamorphosis (Kohl et al., 2013; Longo et al., 2015; Hartmann et al., 2023). Because of these unique amphibian attributes, they are a compelling emerging model system offering information we are unable to acquire from current systems. Therefore, further development and optimization of amphibian systems that make for precise, standardized, and context-independent manipulation of the microbiome will be valuable for future insights on host-microbiome interactions, particularly as they relate to health and disease.
